# Associations of interruptions to leisure-time sedentary behaviour with symptoms of depression and anxiety

**DOI:** 10.1038/s41398-020-0810-1

**Published:** 2020-05-04

**Authors:** Mats Hallgren, Thi-Thuy-Dung Nguyen, Neville Owen, Davy Vancampfort, Lee Smith, David W. Dunstan, Gunnar Andersson, Peter Wallin, Elin Ekblom-Bak

**Affiliations:** 1 Epidemiology of Psychiatric Conditions, Substance use and Social Environment (EPiCSS), Department of Public Health Sciences, Karolinska Institutet, Stockholm, 171 77 Sweden; 2 Behavioral Epidemiology Laboratory, Baker Heart & Diabetes Institute, Melbourne, Australia; and, Centre for Urban Transitions, Swinburne University of Technology, Melbourne, VIC Australia; 3 Department of Rehabilitation Sciences, University of Leuven; and, University Psychiatric Center, Katholieke Universiteit Leuven, Leuven, Belgium; 4 Department of Life Sciences, Anglia Ruskin University, Cambridge, UK; 5 Physical Activity Laboratory, Baker Heart & Diabetes Institute, Melbourne, Australia; and, Mary MacKillop Institute for Health Research, Australian Catholic University, Melbourne, VIC Australia; 6 Research Department, HPI Health Profile Institute, Danderyd, Sweden; 7 Astrand Laboratory of Work Physiology, The Swedish School of Sport and Health Sciences, Stockholm, Sweden

**Keywords:** Psychology, Depression

## Abstract

Interruptions to time spent sitting can ameliorate detrimental metabolic-health consequences of high volumes of sedentary time, but their potential mental health benefits have not been examined. We used the Swedish Health Profile Assessment database, a general health assessment offered to all employees working for companies or organisations connected to occupational and health services. Cross-sectional analyses examined data from 40,550 employees (60% male, mean age = 42 years), collected in 2017–2019. Participants reported the proportion of time (almost always; 75% of the time; 50% of the time; 25% of the time; and almost never) usually spent in leisure-time sedentary behaviours; and, separately, the frequency (never; rarely; sometimes; often; and very often) of interruptions (every 30 min) to sedentary time. Logistic regression models assessed associations of sedentary time, and the frequency of interruptions to sedentary time, with depression/anxiety symptoms. Fully adjusted models included physical exercise. Compared to those in the lowest sedentary time category, those in the medium and high categories had 1.52 (95% confidence interval (CI) = 1.40–1.66) and 3.11 (95% CI = 2.82–3.42) higher odds of frequent depression/anxiety symptoms, respectively. Compared to those who never/rarely interrupted their sedentary time, those who reported interruptions sometimes, often and very often had 0.72 (95% CI = 0.65–0.80), 0.59 (95% CI = 0.53–0.65), and 0.53 (95% CI = 0.46–0.59) lower odds of depression/anxiety symptoms, respectively. In stratified analyses, more frequent interruptions to sedentary time were associated with lower odds of depression/anxiety symptoms, except among those in the lowest interruptions categories (never/25% of the time). More regularly interrupting sitting during leisure-time may reduce the odds of experiencing symptoms of depression and anxiety.

## Introduction

Adults in high-income countries are sedentary (sitting/reclining) for ~8–12 h per day^1,2^. Higher volumes of sedentary time have been linked to greater risk of cardiovascular disease, diabetes, and premature mortality^3,4^, and these associations remain after adjustment for moderate-to-vigorous physical activity^5,6^. The pernicious health effects of this ubiquitous behaviour has led to significant changes in physical activity recommendations, which now recognise the importance of reducing sedentary time in addition to maintaining adequate levels of daily physical activity^3,7^. Potential health benefits of light physical activity, including standing, have also been recognised^8^.

Detrimental associations of sedentary behaviour with major depression and other mental disorders have also been reported^9^. In a meta-analysis, compared to non-occasional/occasional sedentary behaviour, the risk of depression related to the highest categories of sedentary time was 31% higher over 13 cross-sectional studies, and 14% higher over 11 prospective studies^10^. Another review found evidence for a positive association between total sedentary behaviour and the risk of anxiety^11^. Two trials showed that experimentally induced sedentary behaviour can have adverse effects on mood, depression, and inflammatory markers^12,13^. Our recent work indicates that, despite equivalent energy expenditures, sedentary behaviours that are passive in nature (e.g., TV-viewing), and which mainly occur during leisure-time, are more detrimental to psychological wellbeing than mentally active sedentary behaviours (e.g., office work and problem solving), which predominantly occur during work time^14,15^. Preliminary evidence suggests that the latter behaviours may even have beneficial associations with some mental health outcomes^14,16^.

Several studies have examined the impact of interruptions to sedentary behaviour on somatic health outcomes^17^. Such interruptions to sitting time have been variably defined, but generally involve either a standing interruption to extended sedentary time and/or ambulation (i.e., ‘active’ interruption). Interruptions do not necessarily entail structured exercise, but involve moving from a seated to a upright posture. Thus, stretching one’s arms out while remaining seated, for example, would not meet these criteria. A meta-analysis of the relationship between interruptions to sedentary behaviour and cardio-metabolic health found that interruptions of at least light-intensity physical activity may have a positive effect on glycemia in adults^17^. In the same review, pooled analyses from ten observational studies showed a beneficial association of interruptions to sedentary time with obesity metrics that were independent of total sedentary time. In a cross-sectional study, involving 168 middle-aged adults, more frequent interruptions to sedentary time were beneficially associated with waist circumference, body mass index (BMI), triglycerides, and 2-h plasma glucose, independent of total sedentary time and moderate-to-vigorous-intensity activity^18^. Experimental studies have also found robust associations. In a crossover trial, ten overweight/obese adults were exposed to a simulated 9-h laboratory-based sedentary ‘workday’, and completed three brief interruption interventions of varying frequency and duration in a random order^19^. Replacing extended sitting with 2-min of moderate walking every 20 min, or with 2-min of vigorous walking every hour, reduced 18.7-h and postprandial glucose, while 30-min of continuous moderate walking (single session) reduced both glucose and systolic ambulatory blood pressure^19^. Another trial involving 930 middle-aged adults and using accelerometry found that sedentary interruptions were beneficially associated with the metabolic syndrome after adjustment for both moderate-to-vigorous physical activity and fitness^20^.

To our knowledge, no studies have examined associations of interruptions to sedentary behaviour with mental health outcomes. Given the high prevalence of depression and anxiety symptoms in the general population^21^, the established detrimental links between sedentary behaviour and depression^10^, and recent studies indicating potential benefits of interruptions to sedentary time on cardio-metabolic health^17^, exploring these relationships may have important public health and/or clinical implications. We therefore examined associations of leisure-time sedentary behaviour with symptoms of depression/anxiety, and the potential impact of interruptions to sedentary time on these symptoms. A secondary aim was to explore these associations within different strata of sedentary behaviour (low, medium, and high). We hypothesised that more frequent interruptions to sedentary time would be associated with lower odds of depression and anxiety symptoms, and that these associations would be strongest in those with the highest volumes of sedentary time.

## Materials and methods

### Study population

Data originate from the Swedish Health Profile Assessment (HPA) database, managed by the Health Profile Institute (HPI, Stockholm, Sweden: www.hpihealth.se/). HPA includes a one-page questionnaire about lifestyle and health experiences, measurement of anthropometrics and blood pressure, estimation of maximal oxygen uptake from a submaximal fitness test on a cycle ergometer, and a brief dialogue with a HPA ‘coach’ (with qualifications in allied health science) to promote wellbeing. The HPA is offered to employees working for companies or organizations connected to occupational or health-related services. Participation is voluntary and free-of-charge. Although HPA has been running since 1976, we based our analyses on data collected from January 2017 (when specific questions relating to sedentary behaviour were first introduced) to June 2019. The total initial sample comprised 50,264 participants before removal of missing co-variate data (described below). The fully adjusted sample included 40,550 unique cases. Participants were asked to describe their primary occupation in terms of typical daily movement. In total, 53.6% were ‘mostly sedentary with little movement’, 10.3% had occupations that were ‘occasionally physically demanding’, 2.8% were ‘occasionally very physically demanding’, and 20.8% were ‘variable’ (i.e., alternating between sedentary and physically demanding). Thus, more than half the sample had an occupation that was predominantly sedentary. All participants provided informed consent prior to their involvement in the HPA assessment. The study was approved by the Stockholm Ethics Review Board (Dnr 2015/1864-31/2 and 2016/9-32).

### Study outcome: frequent symptoms of depression and anxiety

The primary outcome was assessed with a single question: ‘I experience worry, depressed mood, or anxiety…’ with five response alternatives; very often, often, sometimes, rarely, and never. The first two categories were merged and coded as ‘1’, indicating frequent symptoms of depression and anxiety. The remaining responses were merged and coded as ‘0’ (does not have frequent symptoms of depression/anxiety).

### Exposures

#### Leisure-time sedentary behaviour

This was assessed with the question ‘I sit still during my leisure-time…’ with five response alternatives: Almost always, 75% of the time, 50% of the time, 25% of the time, and almost never. Due to the small proportion of responses to the highest and lowest categories, these were merged into: high (almost always + 75% of the time), medium (50% of the time), and low (almost never + 25% of the time).

#### Interruptions to sitting time

These were assessed with the question ‘Every 30 min, I break up my sitting during leisure-time by at least standing up…’ with five response alternatives: never, rarely, sometimes, often, and very often. The first two categories were merged into one group due to the low number of responses to the first option.

Both questions (above) were adapted from a prospective study of sedentary time and risk of all-cause mortality, cardiovascular disease, and cancer, by Katzmarzyk et al.^5^.

### Covariates

Based on previous evidence of associations of sedentary behaviour with depression^10^, and/or anxiety^11^, the following variables were included in the statistical models.

#### Body mass index

Weight was assessed with a calibrated scale in lightweight clothing to the nearest 0.5 kg. Height was measured to the nearest 0.5 cm using a wall-mounted stadiometer. With these two variables, BMI (kg/m^2^) was calculated, then categorised according to the WHO’s classification for adults; normal weight (<25), overweight (25–<30), and obese (≥30).

#### Smoking

This was assessed by asking participants how often they smoke tobacco (cigarettes), with the response alternatives; ≥20/day, 11–19/day, 1–10/day, occasionally, and never. The first three responses were merged as ‘daily smokers’ (versus occasional and non-smoker).

#### Use of pain medication

This was assessed with the question ‘I use pain medicines…’ with five response alternatives (very often, often, sometimes, rarely, and never). This question was included as a continuous variable.

#### Exercise frequency

This was assessed with the question ‘I exercise/train…’ with eight response alternatives; never, sometimes, one time/week, two times/week, three times/week, four times/week, five times/week, and more than or equal to six times/week.

#### Age and gender

These were self-reported and included as continuous and categorical variables, respectively.

### Statistical analyses

Baseline characteristics of the fully adjusted sample were calculated using descriptive statistics (mean, standard deviation (SD), and percentage %). To assess associations of interruptions to sedentary behaviour with symptoms of depression/anxiety, data were analysed in two steps. First, relationships of leisure-time sedentary behaviour with frequent symptoms of depression/anxiety were explored, using logistic regression and presented as odds ratios (ORs) with 95% confidence intervals (CIs) and *p*-values. To assess a potential dose response, the ‘low sedentary’ category (almost never + 25% of the time) was used as the reference, and compared to the remaining two categories, medium (50% of the time) and high (almost always + 75% of the time). For each association, two models were constructed: crude and adjusted (for age, gender, smoking, use of pain medications, BMI, and exercise frequency). Second, associations of interruptions to sedentary behaviour with frequent symptoms of depression/anxiety were assessed using logistic regression, with the response option ‘never/rarely’ included as the reference category, and compared to the remaining responses (sometimes, often, and very often). These analyses were also run separately within each of the three sedentary behaviour strata. All analyses were performed using R, version 3.5.2.

## Results

### Participant characteristics

In the initial sample consisting of 50,264 people, missing data on co-variates ranged from 0.1% (age) to 13.8% (interruptions to sedentary behaviour). Participant characteristics are shown in Table 1. Participants were middle aged (mean age = 42 years, SD = 12) and there were slightly more males (60.3%) than females. Approximately half of the sample was tertiary educated, and the majority were non-smokers. High durations of leisure-time sedentary behaviour were reported by 14.2% of participants and 8.8% of the sample reported experiencing frequent (often/always) symptoms of depression/anxiety.

**Table 1:** Participant characteristics (*n* = 40,550).

Characteristic	*N* (%)
Age mean (SD); (range)	42.53 (11.90); (16–80)
Gender	
Female	16,093 (39.7)
Male	24,457 (60.3)
Smoking	
Non-smoker	33,836 (83.4)
Occasional smoker	3656 (9.0)
Daily smoker	3058 (7.5)
Exercise frequency	
Never	5099 (12.6)
Sometimes	8467 (20.9)
1–2 times/week	12,463 (30.7)
3–5 times/week	13,084 (32.3)
≥6 times/week	1437 (3.5)
Body mass index (BMI)	
Normal weight	17,332 (42.7)
Overweight	15,459 (38.1)
Obese	7759 (19.1)
Use of pain medications	
Very often	894 (2.2)
Often	1842 (4.5)
Sometimes	8096 (20.0)
Rarely	21,183 (52.2)
Never	8535 (21.0)
Leisure-time sedentary behaviour	
High (always + 75% of the time)	5774 (14.2)
Medium (50% of the time)	15,930 (39.3)
Low (never + 25% of the time)	18,846 (46.5)
Interruption to sedentary behaviour	
Never/rarely	4331 (10.7)
Sometimes	11,832 (29.2)
Often	15,925 (39.3)
Very often	8462 (20.9)
Frequent symptoms of depression/anxiety	
No	36,972 (91.2)
Yes	3578 (8.8)

### Associations of leisure-time sedentary behaviour with frequent symptoms of depression and anxiety

A dose response was observed in both the crude and adjusted models, where increasing volumes of sedentary time were associated with higher odds of depression/anxiety symptoms (Table 2). In the fully adjusted model, compared to those in the low sedentary category, those in the medium and high categories had 1.52 (95% CI = 1.40; 1.66) and 3.11 (95% CI = 2.82; 3.42) higher odds of frequent depression/anxiety symptoms, respectively.

**Table 2:** Associations of leisure-time sedentary behaviour with frequent symptoms of depression/anxiety (*n* = 40,550).

	OR	95% CI	*P*
Crude model			
Low	1	–	–
Medium	1.59	1.46; 1.72	<0.001
High	3.58	3.27; 3.92	<0.001
Adjusted model^a^			
Low	1	–	–
Medium	1.52	1.40; 1.66	<0.001
High	3.11	2.82; 3.42	<0.001

^a^Adjusted for age, gender, smoking, BMI, use of pain medication, and exercise frequency. Low = never + 25% of the time; medium = 50% of the time; high = always + 75% of the time. The total *N* for crude and adjusted models is equal to ensure that the same individuals are compared.

### Associations of interruptions to leisure-time sedentary behaviour with frequent symptoms of depression and anxiety

A dose response was also observed for the exposure ‘interruptions’ to sedentary behaviour. In the fully adjusted model, compared to those who never/rarely interrupted their sedentary time, those who reported interruptions sometimes, often and very often had 0.73 (95% CI = 0.65; 0.80), 0.59 (95% CI = 0.53; 0.65), and 0.53 (95% CI = 0.46; 0.59) lower odds of depression/anxiety symptoms, respectively. Comparison of the crude and adjusted models indicates that inclusion of relevant covariates had minimal attenuating effects on these estimates and their precision.

**Fig. 1:**
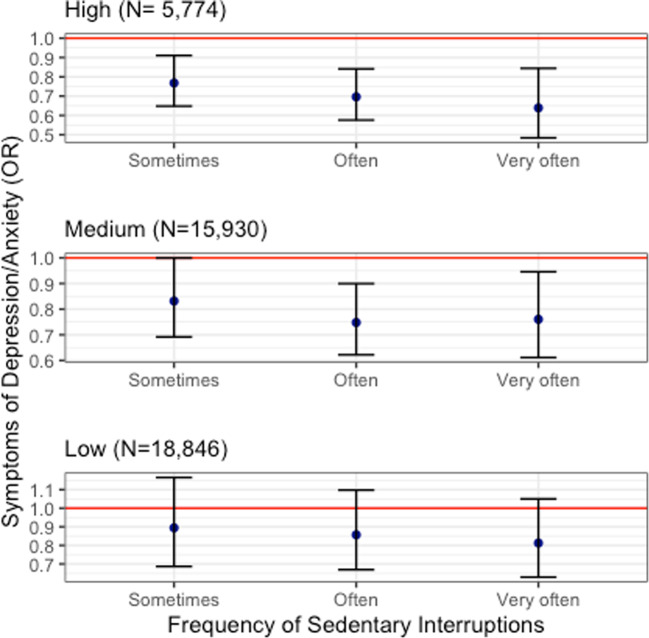
Associations of interruptions to sedentary behaviour with frequent symptoms of depression/anxiety stratified by sedentary behaviour category. All models adjusted for age, gender, smoking, BMI, use of pain medication, and exercise frequency. Reference category = never/rarely. Error bars show the 95% CIs.

Figure 1 illustrates associations of interruptions to sedentary behaviour with frequent symptoms of depression/anxiety, stratified by the three sedentary behaviour categories. Consistent with the pooled analyses, more frequent interruptions to sedentary time were associated with lower odds of depression/anxiety symptoms. However, for those in the low sedentary category (never or 25% of the time), the estimates increased slightly and the associated CIs crossed 1 (Table 3).

**Table 3:** Associations of interruptions to sedentary behaviour with frequent symptoms of depression/anxiety (*n* = 40,550).

	OR	95% CI	*P*
Crude model			
Never/ rarely	1	–	–
Sometimes	0.66	0.60; 0.74	<0.001
Often	0.53	0.47; 0.58	<0.001
Very often	0.47	0.42; 0.53	<0.001
Adjusted model^1^			
Never/rarely	1	–	–
Sometimes	0.72	0.65; 0.80	<0.001
Often	0.59	0.53; 0.65	<0.001
Very often	0.53	0.46; 0.59	<0.001

^a^Adjusted for age, gender, smoking, BMI, use of pain medication, and exercise frequency. The total *N* for crude and adjusted models is equal to ensure that the same individuals are compared.

## Discussion

Interruptions to high volumes of sedentary time are known to be beneficial in the context of markers of metabolic health;^18,19^ this is the first study to examine associations with a mental health outcome of interruptions to sedentary time. Findings indicate that higher volumes of sedentary behaviour in the context of leisure-time increase the odds of self-reported frequent symptoms of depression/anxiety; and, that a higher frequency of interruptions to sedentary time can lower the odds that these symptoms will occur. Stratified analyses were consistent with the pooled data, except among those in the low sedentary category. This suggests that the potential mental health benefits of interrupting sedentary behaviour may be greater in those who are highly sedentary. Previous research has demonstrated that interruptions to sedentary time can improve cardio-metabolic health^17,18,22^; however, there is a dearth of evidence from studies investigating potential mental health impacts of such interruptions. While there is evidence from prospective observational studies and recent trials showing that higher volumes of sedentary behaviour can be associated with depressive symptoms and related mood disorders^10,13^, a more nuanced understanding of the patterns of sedentary time could help to inform public health guidelines and clinical treatment options.

Although no previous studies have examined associations of interruptions to sedentary behaviour with depression or anxiety, links with cognitive functioning have been reported. These studies are relevant, as depression is associated with cognitive deficits that can contribute to attentional biases, rumination, and worsened mood states, while physical activity is shown to benefit cognition in those with depression^23,24^. In a randomised cross-over trial, Wheeler et al. compared the effects of a morning bout of moderate-intensity exercise, with and without subsequent light-intensity walking breaks from sitting, on cognition in older (mean age = 67) overweight/obese adults^25^. Working memory and executive functioning improved in the exercise plus ‘breaks’ condition relative to extended sitting only, while serum BDNF increased in both the exercise only and exercise plus breaks condition, compared to extended sitting^25^. Changes in cerebral blood flow could underlie the putative benefits of interruptions to sedentary time on cognition and mental health. Decreased cerebrovascular blood flow and function are associated with worse cognitive functioning in depression^26^, while prolonged sitting can impair peripheral blood flow and function^27^. One study explored the effect of breaking up extended sitting on cerebrovascular blood flow in healthy desk workers^28^. Uninterrupted sitting was shown to reduce cerebral blood flow; however, this reduction was offset when frequent (every 30 min) short walking breaks were incorporated into the sitting period. Interruptions to prolonged sitting could also improve feelings of fatigue—another common symptom of depression^29^. Wennberg et al. compared the acute effects of uninterrupted sitting (2 h) with sitting interrupted by brief (3 min) bouts of light-intensity walking on self-reported fatigue^30^. During the ‘breaks’ condition, fatigue levels were lower at 4 and 7 h post intervention, respectively, compared to the sedentary condition.

Sedentary behaviours could also displace time spent in physical activity, which is shown to reduce the risk of depression. Another hypothesis is that extended sedentary time involves socially isolated activities (e.g., TV-viewing), which may remove people from mood-enhancing social interactions. Evidence suggests that depression can be linked to higher levels of inflammatory markers^31^, and one trial has shown that experimentally induced sedentary time was associated with higher interleukin-6 levels in response to stress^13^. A systematic review of 25 interventions found evidence that uninterrupted sedentary behaviour results in moderate and deleterious changes in insulin sensitivity, glucose tolerance, and plasma triglyceride levels^32^. A prospective study found associations between sedentary behaviour and increases in various acute phase reactants and coagulation markers in older adults over 4 years. Preliminary evidence suggests that glycemic variability may influence brain health and cognition^33^. As sedentary behaviour frequently involves the use of screen-based devices, these activities could also contribute to sleep and mood disorders. Moreover, because sedentary behaviour primarily occurs indoors, away from direct sunlight, the possibility that these behaviours might reduce vitamin D exposure, which in turn is shown to affect mood symptoms, also warrants exploration.

We focused on sedentary behaviours occurring during leisure-time, as our ongoing work suggests that these behaviours can be more detrimental to mental health than those occurring during work time^34^. In two recent studies, we have made a distinction between sedentary behaviours that are mentally ‘passive’ (e.g., TV-viewing) and those that are mentally active (e.g., reading and office work)^14,15^. While both behaviours involve low energy expenditures (METs), they are uniquely characterised by different levels of cognitive effort, with the latter involving more. In both studies, longer durations of passive sedentary behaviour were found to increase the risk of depression, while mentally active sedentary behaviours were protective against depression^15^. Leisure-time may involve both passive and mentally active sedentary behaviours; however, recent studies indicate that TV-viewing constitutes a large proportion of total leisure-time sedentary behaviour among adults^35,36^.

From a clinical perspective, our findings suggest that interrupting sedentary behaviour in leisure contexts with regular physical activity (including standing) could have mental health benefits, and should therefore be promoted in those with depression/anxiety symptoms, in addition to maintaining recommended physical activity levels. Given the established cardio-metabolic benefits of interrupting extended sitting^17^, and the mechanisms that underlie both cardio-metabolic and mental health^37^, the current findings may also have relevance for the prevention of mood disorders. In a recent study, we observed that sleep quality mediated associations of extended passive sedentary behaviour with depression^38^. Other lifestyle factors, including diet and stress, may also play a role and warrant further investigation.

Interruptions to sedentary time in domestic and leisure settings could be achieved through different strategies. A simple message that is both practical and consistent with epidemiologic evidence^39^, would be to stand up and move after 30 min of uninterrupted sitting. Light-intensity activities could be encouraged to interrupt sedentary time (e.g., standing up while talking on the phone or during TV commercials). Reviews of intervention studies suggest that a multi-component approach may be beneficial^36,40^. This might consist of behaviour change techniques based on motivational interviewing and goal setting;^41^ the use of mobile phone apps shown to reduce sedentary behaviour in leisure settings^42^; and information to increase knowledge of the independent effects of sedentary behaviour and physical activity on wellbeing. However, in one trial, four individual theory-based counselling sessions delivered over 6 months did not increase the number of sedentary interruptions^43^, suggesting that counselling may need to be complemented with other strategies, such as electronic reminders to stand up and move. Another trial utilising a combination of face-to-face counselling sessions, phone calls and newsletters reported significant increases in the number of steps taken during TV commercials over 6 months^44^. Thus, both the frequency and type of contact with study participants appears to impact intervention adherence.

Strengths of the study include the large participant sample, and the unique inclusion of a question to assess interruptions to sedentary behaviour. The main study limitation is the cross-sectional design, which does not enable the direction of these relationships to be established. The findings of a recent Mendelian Randomisation study suggests that sedentary behaviour may be causally related to depression^45^; experimental studies are a logical next step in testing these relationships. The sedentary interruptions question specified the frequency (every 30 min) but not the duration of the interruptions. This is a possible limitation, as the duration of these interruptions could (potentially) impact mental health outcomes. The exposures and outcome were self-reported, not objectively measured; thus, both sedentary behaviour and depression/anxiety symptoms may have been underestimated. Moreover, the use of antianxiety and antidepressant medication was not assessed in the questionnaire, which meant we could not adjust for possible differences in psychotropic drug use. Finally, some residual confounding may exist, as socio-economic status (i.e., income and education) were not directly assessed in the survey, and therefore could not be included as covariates in the analyses.

In conclusion, these findings suggest that higher volumes of sedentary behaviour in leisure-time contexts increase the odds of frequent symptoms of depression and anxiety, while frequent interruptions to extended sitting/reclining in this context may reduce the odds that these symptoms will occur. Prospective studies of these relationships are needed, preferably using objective measures of sedentary behaviour. Future trials could examine effects on depression following interventions to reduce total sedentary time and increase the number of interruptions to sedentary behaviour. These strategies could be compared to conventional aerobic or strength training interventions that have previously been shown to be efficacious^46^. A better understanding of the neurobiology underlying relationships of sedentary behaviour and depression/anxiety is also needed. Neuroimaging techniques and blood analyses could explore speculative mechanisms, including neurogenesis, blood flow changes, and glycemic variability^47^.
